# Mitochondrial pH Nanosensors for Metabolic Profiling of Breast Cancer Cell Lines

**DOI:** 10.3390/ijms21103731

**Published:** 2020-05-25

**Authors:** Consuelo Ripoll, Mar Roldan, Rafael Contreras-Montoya, Juan J. Diaz-Mochon, Miguel Martin, Maria J. Ruedas-Rama, Angel Orte

**Affiliations:** 1 Departamento de Fisicoquimica, Facultad de Farmacia, Unidad de Excelencia en Quimica Aplicada a Biomedicina y Medioambiente (UEQ), Universidad de Granada, Campus Cartuja, 18071 Granada, Spain; consueloripoll@ugr.es (C.R.); mjruedas@ugr.es (M.J.R.-R.); 2 GENYO, Pfizer-Universidad de Granada-Junta de Andalucia Centre for Genomics and Oncological Research, Avda Ilustracion 114, PTS, 18016 Granada, Spain; mar.roldan@genyo.es (M.R.); juandiaz@ugr.es (J.J.D.-M.); miguelmartin@ugr.es (M.M); 3 Departamento de Quimica Organica, Facultad de Ciencias, Unidad de Excelencia en Quimica Aplicada a Biomedicina y Medioambiente (UEQ), Universidad de Granada, Campus Fuentenueva, 18071 Granada, Spain; rcm@ugr.es; 4 Departamento de Quimica Farmaceutica y Organica, Facultad de Farmacia, Unidad de Excelencia en Quimica Aplicada a Biomedicina y Medioambiente (UEQ), Universidad de Granada, Campus Cartuja, 18071 Granada, Spain; 5 Departamento de Bioquimica y Biologia Celular I, Facultad de Ciencias, Universidad de Granada, Campus Fuentenueva, 18071 Granada, Spain

**Keywords:** FLIM microscopy, nanosensing, intracellular sensors, tumoral metabolism, cancer metabolism

## Abstract

The main role of mitochondria, as pivotal organelles for cellular metabolism, is the production of energy (ATP) through an oxidative phosphorylation system. During this process, the electron transport chain creates a proton gradient that drives the synthesis of ATP. One of the main features of tumoral cells is their altered metabolism, providing alternative routes to enhance proliferation and survival. Hence, it is of utmost importance to understand the relationship between mitochondrial pH, tumoral metabolism, and cancer. In this manuscript, we develop a highly specific nanosensor to accurately measure the intramitochondrial pH using fluorescence lifetime imaging microscopy (FLIM). Importantly, we have applied this nanosensor to establish differences that may be hallmarks of different metabolic pathways in breast cancer cell models, leading to the characterization of different metabophenotypes.

## 1. Introduction

The intracellular equilibrium of hydronium ions is finely controlled by proton pumps and directly involved in cell metabolism. Although the pH of the cell is regulated, some factors or processes can lead to alterations, which sometimes may be related to disease. Cellular pH can also be affected by cellular processes such as senescence or programmed cell death, known as apoptosis. In addition, the presence of exogenous substances in the cell, such as drugs, can modify the chemical composition of the medium, causing a variation in the cellular pH. For instance, cancer cells have an adaptation of their metabolism, known as the Warburg effect [1,2]. In proliferating or cancerous cells, most of the pyruvate resulting from glycolysis is converted into lactate. This lactate is transported to the cellular exterior by monocarboxylate transporters, thus altering the pH of the culture medium where the cells grow.

Moreover, mitochondria are cellular organelles that carry out different functions, the most remarkable of which is the production of cellular energy (ATP) through an oxidative phosphorylation system. Oxidative phosphorylation involves the coupling of redox and phosphorylation reactions in the inner membrane of mitochondria. During this process, the electrons of NADH or FADH are transported through the electron transport chain, creating a proton gradient. The movement of protons from the mitochondrial matrix to the intermembrane space creates a pH gradient and an electrical gradient that drive the synthesis of ATP [3]. There are many diseases that occur as a result of incorrect functioning of mitochondria, such as Leigh syndrome [4], mitochondrial encephalomyopathy [5], lactic acidosis, stroke-like episodes, hepatopathy, tubulopathy [6,7,8], and cancer [9,10]. Hence, it is of utmost importance to understand the relationship between mitochondrial pH (pH_m_), tumoral metabolism, and cancer.

To achieve this goal, we aimed to develop a nanotechnological sensing platform to provide accurate readouts of the intramitochondrial pH in different cancer cell lines. While there are different approaches for sensing inside living cells, obtaining robust and accurate results is not a trivial matter. Most of the intracellular sensing approaches are based on the use of fluorescence microscopy, especially ratiometric sensors, which exhibit changes in the ratio between luminescence emission intensity at two different wavelengths that are correlated with the presence of the analyte of interest. Although the use of ratiometric luminescent sensors is quite widespread, being used, for instance, in fluorescence resonance energy transfer (FRET), this approach is not free from systematic errors. Important uncertainties may arise when one of the bands is affected by other sources within the cellular environment (quenching, photobleaching, and so forth) or by the presence of emissive interferences, such as the species causing cellular autofluorescence (i.e., structural fluorescent groups such as tryptophan and tyrosine in proteins, lipofuscin in cell membranes, NADH in the cytosol, porphyrins in the blood, albumin in serum, B vitamins, etc.), which may alter the sensing ratio [11,12].

One way to overcome many of these limitations is by using time-resolved fluorescence spectroscopy. This family of luminescence techniques yields the photoluminescence (PL) lifetime, τ, an average measure of the time for which a system remains in the electronically excited state upon excitation. τ is a purely kinetic parameter related to all deactivation processes occurring in the excited state. Time-resolved fluorimetry, combined with fluorescence microscopy, is known as fluorescence lifetime imaging microscopy (FLIM). FLIM is inherently multidimensional since not only the PL emission intensity but also the PL lifetime values in each pixel of the image is collected, usually represented in pseudocolor image scales, serving as an extra contrast tool. Importantly, FLIM is especially suited for the quantitative real-time detection of biologically important molecules or ions within living cells [12]. The PL lifetime is a parameter that has numerous advantages, such as being independent of the fluorophore concentration and the excitation power, in addition to possible heterogeneities in the optical properties of the medium in which it is located. Although FLIM is less exploited for intracellular sensing than its ratiometric counterparts, there are many recent examples of FLIM-based sensors, many of which are based on FRET [13] for the detection of pH changes [14,15], specific proteins such as Ras [16], or tyrosine kinase Src [17]; on molecular rotors for the detection of environment and cellular viscosity [18,19,20]; or on the aggregation state of dyes inside cellular mimicking structures [21]. However, many of these works employ organic fluorophores or fluorescent proteins, variants of Green Fluorescent Protein (GFP), as the emissive species. In general, the fluorescence lifetimes of organic fluorophores and GFP variants are usually in the range of 1–5 ns [13]. These relatively short lifetimes provide limited sensitivity in terms of the variation range that the sensing mechanism may produce. Likewise, these values are in the same range as that of cellular autofluorescence, thus not avoiding potential interferences from the cell’s inherent luminescent components.

Unlike organic fluorophores, quantum dot (QD) nanoparticles show unique properties in terms of their PL lifetime. Normally, QDs have long lifetimes (between five and hundreds of nanoseconds) and multiexponential PL decays. In addition, the lifetimes of QDs are significantly longer than the autofluorescence decay of cells (2–3 ns) and the fluorescence lifetimes of conventional dyes (1–5 ns), but efficient enough to maintain a high flow of photons [22]. Therefore, the combination of time-resolved luminescence techniques with QD-based nanosensors is an attractive approach, especially for intracellular application, allowing the determination and monitoring of changes in the concentrations of molecules and ions of biological interest in the cell interior during important physiological processes. For example, our research group has developed QD-based nanosensors for chloride ions [23] and zinc ions [24] and a pH nanosensor [25,26] particularly designed to be used intracellularly to provide quantitative information through FLIM. Therefore, our interest in developing a QD-based, intramitochondrial pH nanosensor, involves two requirements: making the QD pH-responsive and being selectively delivered into the mitochondria. While QD pH nanosensors can be obtained by modifying the QD surface with thioalkyl-carboxylates, such as mercaptoacetic acid, mercaptopropionic acid (MPA), and mercaptosuccinic acid [25,26,27,28,29], thus that the PL properties are sensitive to the degree of protonation of the carboxylic acid [30], addressing them into mitochondria has not been reported yet.

In this manuscript, we present QD that are pH-responsive and mitochondrial-targeted. We take advantage of the long PL lifetime of QDs to develop intramitochondrial nanosensors to quantitatively report the pH_m_ values inside these organelles utilizing FLIM. As proof of concept, we have used this method to establish differences that may be hallmarks of different metabolic pathways in breast cancer cell models. Breast cancer cell models present a series of features that make them ideal for this study. These models are well established from a clinical and immunological point of view. They are clinically classified into five subtypes depending on their location and immunological profile. We hypothesize that the differences in the clinical classification could also lead to differences in the metabophenotypes and hence feasible detection using nanosensors and FLIM imaging.

## 2. Results

### 2.1. QD-SS-MPA Nanosensor Preparation, Characterization, and Calibration

**Figure 1:**
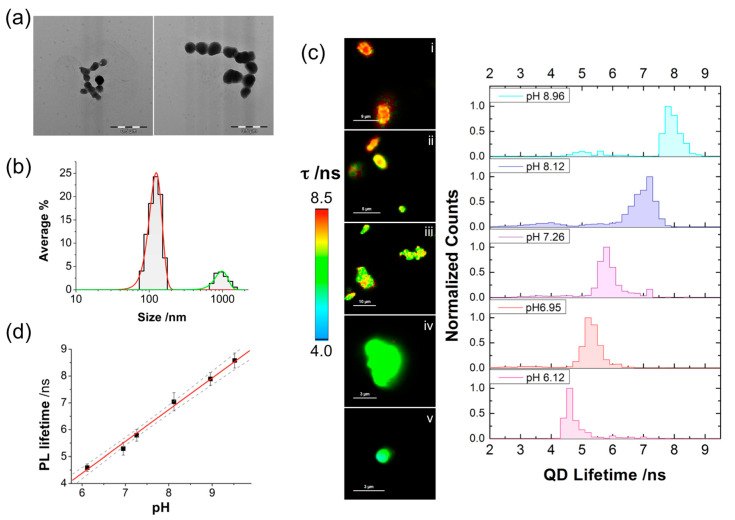
(**a**) TEM images of QD-SS20-MPA. Scale bars correspond to 0.5 µm. (**b**) The particle size distribution of QD-SS20-MPA suspended in phosphate buffer at pH 8.0. The distribution is the average of three independent measurements. (**c**) Representative fluorescence lifetime imaging microscopy (FLIM) images and photoluminescence (PL) lifetime distributions of QD-SS20-MPA nanosensors on glass slides suspended in intracellular cell buffer at different pH values: (i) 8.96; (ii) 8.12; (iii) 7.26; (iv) 6.95; and (v) 6.12. The images were selected from a set of 10 different images at each pH. Scale bars correspond to 9 (i), 5 (ii), 10 (iii), 3 (iv), and 3 µm (v). The corresponding normalized PL lifetime distributions are the average of 10 different pictures of different fields of view. (**d**) PL lifetime versus pH calibration obtained from FLIM images from QD-SS20-MPA nanosensors on glass slides. Black squares represent the center of the PL distributions, as obtained by fitting the distribution to a Gaussian function, and error bars represent the associated error of the fitting. The red line is the linear fitting of the data (*r*^2^ = 0.994), whereas the dashed lines represent upper and lower confidence limits of the fitting at 95% confidence.

The design of the nanosensor involved a double modification of the QD surface. On the one hand, the nanoparticles included MPA as a pH-sensitive group, which affects the PL lifetime of the QDs [26]. On the other hand, Szeto-Schiller (SS) peptides were added to the surface for specific mitochondrial delivery [31]. The preparation conditions were finely optimized to maintain the pH response of the PL properties and avoid excessive coalescence of the nanoparticles. Three different conjugates modified with three different SS peptides were prepared and tested: QD-SS20-MPA, QD-SS02-MPA, and QD-SS31-MPA. For the conjugation of the different SS peptides with the QDs, we synthesized modified versions of the SS peptides containing an 11-carbon aliphatic chain ending with a thiol group at their N-terminal end. Conjugation with the QD surface occurred via formation of self-assembly monolayers between thiol groups and metallic surfaces (see Supplementary Materials, SM, Schemes S1–S4 and Table S1 for details on the synthesis of the modified SS peptides and their sequences). Following conjugation of the SS peptides to the QDS, these were incubated with MPA. Further details on the preparation of the QD-SS-MPA nanosensors can be found in the Experimental section. The initial pH sensing and cell delivery tests with the new materials demonstrated that the only useful nanosensor was QD-SS20-MPA. The QD-SS02-MPA nanoparticles did not show an adequate response towards pH, whereas the QD-SS31-MPA nanoparticles had a good response to pH but did not penetrate into the mitochondria (Figure S1). Hence, we focused on the use of only QD-SS20-MPA. The QD-SS20-MPA nanoparticles were characterized by dynamic light scattering (DLS) and electron microscopy (TEM) (Figure 1a,b). While they showed a certain degree of aggregation, there were a large number of individual nanoparticles. The DLS measurements reported the main population of nanoparticles 124 ± 25 nm in size (the error was reported as the standard deviation of the population based on Gaussian function fitting) and a secondary population of aggregated species with a size of 990 ± 200 nm.

To apply these nanoparticles as FLIM nanosensors, the first step was to calibrate the pH response of the QD-SS20-MPA conjugate in complex media. FLIM images of the emission of QD-SS20-MPA deposited on glass slides and suspended in media mimicking the cellular environment at different pH values allowed us to calibrate the response using the PL lifetime, τ, as the sensing parameter. Figure 1c shows representative FLIM images and PL distributions of these nanosensors. Using the average of τ distributions from six images at each pH value, we built a calibration response plot Figure 1d, which exhibited a linear relationship between the average PL, τ, and the pH. We then established the calibration equation τ /ns = 1.214·pH – 2.965, which would allow us to determine the pH value of the medium surrounding the QD nanosensor via measurements of the PL lifetime in FLIM imaging.

### 2.2. QD-SS20-MPA Mitochondrial Localization

The next step was to test the effective localization of the QD-SS20-MPA nanosensors inside mitochondria of different cell lines, specifically breast cancer cells. In this work, we chose an osteoblast cell line, 143B, and four different breast cancer cell lines, according to the current clinical classification [32]: MCF7, ZR751, MDA-MB-231, and MDA-MB-468. MCF7 and ZR751 are cell lines representative for a clinical subtype featuring a hormone receptor-positive expression profile, non-invasive, and low proliferating cells, which correlated with a less aggressive, and good prognostic clinical outcome. The other two cell lines, MDA-MD-231 and MDA-MD-468, are representative for a triple-negative (hormone receptor and HER2 growth factor expression) clinical subtype, which correlates with an invasive-high proliferating profile with a poor prognostic outcome. The cell lines were cultured on glass slides for 2 days, and then the intramitochondrial pH nanosensor was added to the culture medium. The cell lines were incubated for 2 h to allow the nanosensor to enter the cells and find the mitochondria. Then, the mitochondria-staining dye MitoTracker Deep Red (MT) was added to the medium. We used dual-color FLIM-pulsed interleaved excitation (PIE) [33] to follow the localization of the QD-SS20-MPA nanosensor and to verify the colocalization with mitochondria.

**Figure 2:**
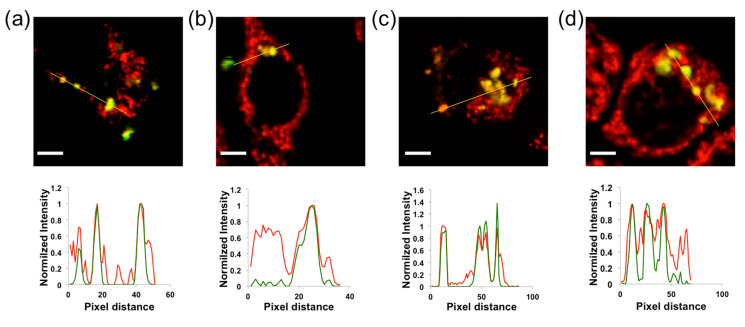
Representative colocalization images, selected from a set of at least 15 different images in each case, of the QD-SS20-MPA nanosensors (green channel) and the MT staining dye (red channel) in 143B osteoblasts (**a**), and MCF7 (**b**), MDA-MB-231 (**c**), and MDA-MB-468 (**d**) breast cancer cells. The intensity profiles correspond to the yellow lines drawn in the images. The scale bars represent 10 µm.

Figure 2 and Figure S2 (in the SM) show representative examples of images of QD-SS20-MPA nanosensors in different breast cancer cell lines stained with MT. Intensity plots correspond to lines drawn in yellow in each image. Usually, the QD-SS20-MPA nanosensors were not distributed along all mitochondria, but they covered some of them. Due to this behavior, we quantified the level of colocalization as the percentage of colocalized QD-SS20-MPA pixels (green channel) and obtained (93 ± 3)% for 143B, (79 ± 11)% for MCF7, (98 ± 3)% for MDA-MB-231, and (97 ± 5)% for MDA-MB-468 cell lines. These results demonstrated that the QD-SS20-MPA nanosensors were successfully delivered to the mitochondria, and very good colocalization was obtained in the 143B, MCF7, MDA-MB-231, and MDA-MB-468 cell lines. In the case of ZR751, the QD-SS20-MPA nanosensors were not uptaken by cells. Since our main interest lies in comparing the metabolic features of breast cancer cells, our results in the next section are limited to the three breast cancer cell lines in which the nanoparticles were successfully delivered into the mitochondria. Moreover, the QD-SS20-MPA dose employed for imaging did not show remarkable cell toxicity to the cells, as evidenced by cell viability measurements (see Figure S4). 

### 2.3. Intramitochondrial pH_m_ Estimation through FLIM Imaging

**Figure 3:**
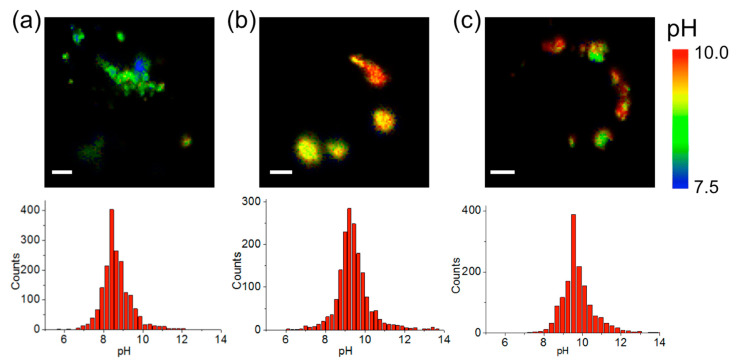
Representative pH_m_ images and the corresponding pH_m_ distributions obtained from the PL lifetime, *τ*, of QD-SS20-MPA nanosensors incorporated into the mitochondria of MCF7 (**a**), MDA-MB-231 (**b**), and MDA-MB-468 (**c**) breast cancer cells. Scale bars represent 10 µm.

After calibration and testing of the efficiency for mitochondrial delivery were performed, FLIM images from QD-SS20-MPA nanosensors incorporated into breast cancer cells were obtained. At least 20 different images from different nanosensor preparations and different cell cultures were collected per cell line, ensuring the robustness and reproducibility of our results. To focus on the mitochondrial pH_m_, the pixels of interest were selected as those in which colocalization of the QD-SS20-MPA luminescence was coincident with MT fluorescence emission in the red channel. Then, the average PL *τ* of the nanosensor in such pixels was converted into pH_m_ using the calibration equation. Figure 3 and Figure S3 (SM) show representative pH_m_ images from the QD-SS20-MPA sensor inside mitochondria of different cell lines. Interestingly, a certain degree of pH_m_ heterogeneity between different mitochondrion was visible in these images (Figure 3 and Figure S3). This may be related to the ubiquitous photon noise distribution, as photon emission is a random event, but it may be caused by actual differences in pH_m_. In fact, mitochondrial pH alkalinization spikes were found to dynamically respond to metabolism and signaling events [34,35]. However, these spikes do occur within 60–70 s time-frames, and, unfortunately, our instrumental settings required slow scanning rates to ensure high photon counts for accurate lifetime determination. Hence, dynamic changes in mitochondrial pH_m_ in such time scale would have been hidden during scanning, but our results would directly show a snapshot of a specific moment.

**Figure 4:**
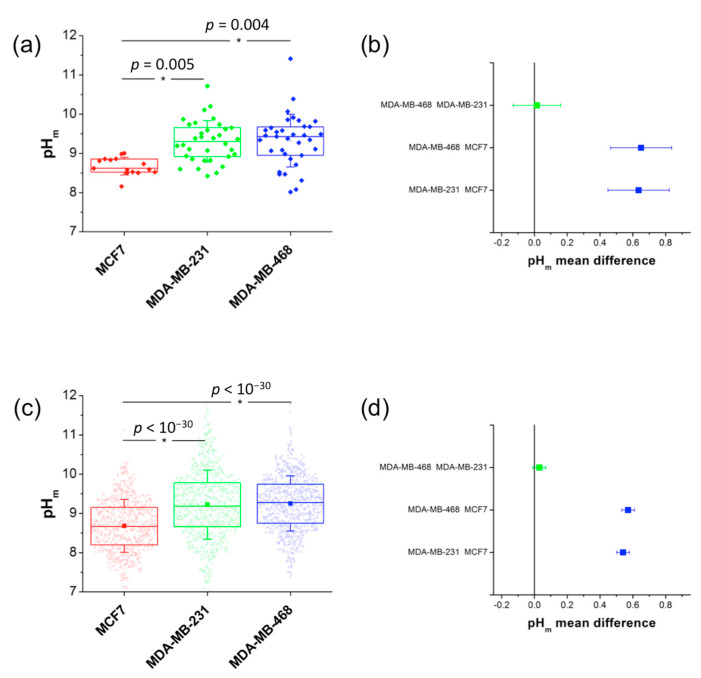
(**a** and **c**) Box plot of the pH_m_ distributions obtained in different image repetitions (**a**) and obtained pixelwise (**c**), where the box includes 75% of the images or pixels, respectively, and the whiskers represent the standard deviations. (**b** and **d**) Differences in the means of the pH_m_ distributions obtained in different image repetitions (**b**) and obtained pixelwise (**d**). The distributions were compared using the Bonferroni statistical test at a significance level of 0.01. The asterisks in panels (**a**) and (**c**) indicate that the marked distribution is significantly different from the other two. In panels (**b**) and (**d**), blue squares indicate significant differences, whereas green squares represent nonsignificant differences.

Our results, averaged over at least 20 different images in different repeated experiments with different nanosensor batches, showed a pH_m_ value of 8.67 ± 0.06 (error stated as the standard error of the mean, SEM) for MCF7 cells, 9.31 ± 0.09 (SEM) for MDA-MB-231 cells, and 9.32 ± 0.11 (SEM) for MDA-MB-468 cells. The overall pH_m_ distributions from all the collected images were compared to check whether the differences between the obtained pH values were significant. For this purpose, the Bonferroni statistical test was performed, with a significance level of 0.01 (Figure 4). Interestingly, the MCF7 cell line displayed a significantly lower pH_m_ value in the mitochondrial area than did the triple-negative cell lines (MDA-MB-231 and MDA-MB-468). Previously reported pH_m_ values of 8.0 ± 0.1 for MCF7 cells [36,37], and in other cell types [38,39], were lower than our findings (see Discussion). In any case, our method allowed the identification of significantly larger pH_m_ values for the triple-negative cell lines compared to that in MCF7 cells.

### 2.4. Correlation with Different Metabolic Features

**Figure 5:**
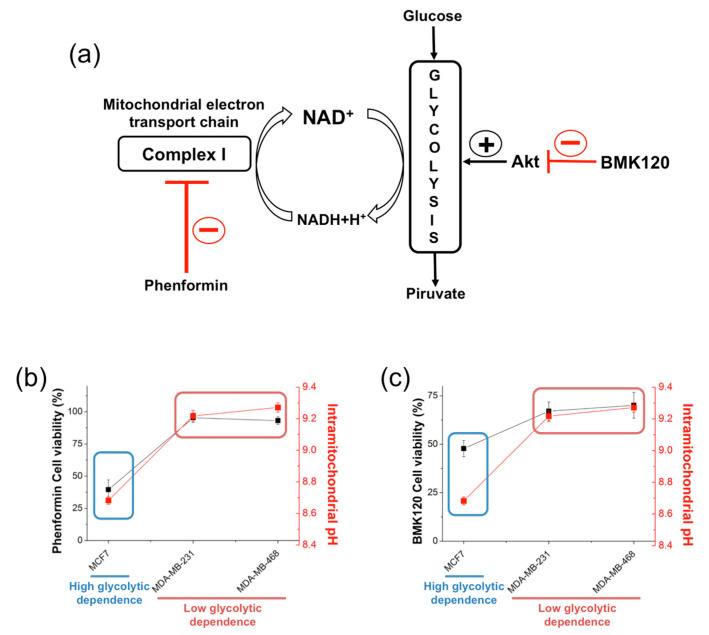
(**a**) Regulatory mechanism for the effect of BMK120 and phenformin. (**b** and **c**) Correlation of the mitochondrial pH (red) with cell viability upon treatment with BMK120 (**b**) and phenformin (**c**) in the MCF7, MDA-MB-231, and MDA-MB-468 breast cancer cell lines. This correlation clearly highlights two different metabolic behaviors with high (MCF7) or low (MDA-MB-231 and MDA-MB-468) glycolytic dependence.

We found that the MDA-MB-231 and MDA-MB-468 cell lines showed higher intramitochondrial pH values than the MCF7 cell line. The statistically significant differences in pH_m_ could be attributed to distinct metabolic features. Hence, we tested the correlation of the results obtained with our pH_m_ nanosensors in living tumor cells with actual metabolic behaviors. We hypothesized that different values in pH_m_ may be related to differences in glycolytic activity and mitochondrial activity. We carried out experiments using metabolic inhibitors, aiming to answer whether the overall metabolism of these cells mainly relies on a high glycolytic dependence. Accordingly, two compounds were selected, phenformin, which is a mitochondrial respiration complex I inhibitor, and BMK120, which is a specific inhibitor of the Akt oncogenic kinase (Figure 5a). Phenformin inhibits the reduction of coenzyme Q in the electron transport chain, therefore avoiding the oxidation of NADH to NAD+ in the respiration complex I. Thus, phenformin inhibition provokes a breakdown in the NAD^+^ balance, which is fundamental to sustain an active glycolytic flux, since NAD^+^ is consumed by glycolityc enzymes, and requires permanent recycling to sustain a fully active pathway (Figure 5a). On the other hand, BMK120 inhibition of Akt likely induces a collapse on glycolytic flux, since Akt is a main activator of several enzymes of the glycolytic pathway (Figure 5a) [40,41].

Interestingly, our results clearly displayed two distinct glycolytic phenotypes, so-called metabophenotypes. MCF7 cells displayed a stronger dependence on glycolytic pathways, since both, NAD^+^ imbalance due to phenformin inhibition on respiration complex I (Figure 5b) and Akt inhibition due to BKM120 treatments (Figure 5c) provoked a significant loss of cell viability. In contrast, MDA-MD-231 and MDA-MB-468 resulted almost resistant to drug treatments, thus suggesting a low glycolytic dependence. Of note, pH_m_ measurements by using the intramitochondrial-delivered pH nanosensors strikingly correlated with the metabophenotypes observed for the drug-induced glycolytic collapse experiments.

## 3. Discussion

In this work, we used a nanotechnological approach to directly probe the intramitochondrial pH. For this purpose, we designed and optimized different nanosensors by incorporating SS peptides as mitochondria-targeting groups on the surface of the nanoparticles and MPA as pH-responsive moiety.

Optimization of the nanosensing platform was difficult, as many approaches did not work out as expected, especially when the SS peptides were added onto the surface. An important consideration for the performance of our pH_m_ nanosensors is the actual localization of the sensor within the mitochondrial matrix. SS peptides are known to interact with the mitochondrial inner membrane, usually though to occur via a specific interaction with cardiolipin [31,42], a phospholipid enriched in such membrane. However, very recently, Mitchell and colleagues reported that the interaction of SS peptides with the inner mitochondrial matrix does occur through surface charge interactions, and mentioned that ongoing efforts are being taken to probe the distribution of SS peptides in the mitochondrial matrix-exposed face of the inner membrane [43]. The interaction of SS-31 with the mitochondrial inner membrane is widely studied [42]. However, studies directly probing the interaction of cardiolipin with SS-20 are scarce. Interestingly, while QD-SS20-MPA nanosensors exhibited clear mitochondrial localization, the QD-SS31-MPA analogs did not (see Figure 2, Figures S1 and S2). We hypothesize that this differential behavior is caused by the redox properties of the peptides. As it is well known, SS-20 is less easily oxidized than SS-31 [44], thus that charge transfer to the CdSe/ZnS is feasible and facilitated by the ZnS layer [45]. Therefore, we believe that charge transfer between the QD and the SS-31 peptide alters the mitochondrial targeting capabilities of the peptide. Regarding the actual localization of the QD-SS20-MPA nanosensor, its interaction with the external part of the cardiolipin-rich crystae would entrap the nanosensors in the intermembrane space, instead of the mitochondrial matrix. The optical resolution of our instrument is defined by the diffraction limit, thus that it did not allow confirmation of the presence of the sensors within the matrix, but in our ongoing research, we are employing super-resolution nanoscopy to gain further insights. In any case, given that mitochondria crystae are actually proton traps in the intermembrane space [46], it is not likely that the high pH values that we measured corresponded to such space. Furthermore, previous experiments by Horton and colleagues [47] using fluorescently labeled SS-20 supported that binding to the internal membrane was not the only mechanism for mitochondrial delivery and that the mitochondrial matrix was accessible given the response of the labeled peptide to mitochondrial depolarization. Therefore, although actual localization of our nanosensor was not possible, there exists supporting evidence that they do report on the alkali environment of the mitochondrial matrix.

Once the mitochondrial localization and response of the QD-SS20-MPA nanosensor was optimized, the advantages of FLIM were key to providing quantitative information with high spatial resolution. Previous studies have suggested the importance of pH_m_ as a key metabolic parameter, combined with the mitochondrial membrane potential [48]. In previous literature, ratiometric pH sensors have been employed, directly targeting mitochondria. Initial reports employed ratiometric, fluorescent-protein-based pH sensors, although several problems are associated with these techniques, such as differences in expression and folding levels [49]. More recently, ratiometric sensors containing organic fluorophores and a mitochondria-targeting group, such as triphenyl phosphine (TPP), have been reported [36,37,50]. Nevertheless, these approaches are not free of the inconveniences of ratiometric methods. For instance, a methodology was proposed in which the intensity ratio was calculated using the emission of a TPP-modified quinolimide-based probe and a red MitoTracker dye [50]. As the study is based on the individual emissions of the two probes, the methodology assumes that the two dyes penetrate the mitochondria in the same proportion, but this assumption may not always be true, leading to significant systematic errors in the pH determination, which may be related to the large fluctuations found in pH_m_, ranging between 4.63 and 8.05. Likewise, changes in the emission properties of the MitoTracker emission intensity due to pH changes or photobleaching were not considered, with two factors causing alterations in the measured ratio. Other examples of ratiometric pH sensors employed inside mitochondria were based on modified hydroxypyrene [36] and fluorescein [37]. In both cases, the presence of excited-state proton transfer reactions may alter the readouts of the ratio, and accurate quantitative information determined by these methods may not be entirely robust, unless a careful consideration of all the excited-state dynamics, contribution of autofluorescence, and photobleaching rates are performed [39].

FLIM-based quantitative sensors hold many advantages over conventional ratiometric methods, although they are not free from certain disadvantages [12,51]. Among such disadvantages, an important factor in our work was the requirement for long acquisition times [51], in order not to cause photon pile-up. This prevented us from exploring real-time dynamics in pH_m_ values as detected with other methods [34,35]. Novel fast-FLIM instruments and methodologies are currently available [52], thus that there is still room for improvement in the results that we can obtain with our nanosensors in the future.

Previous works focusing on pH_m_ measurements explored relative changes related to interesting physiological events; for instance, the acidification of mitochondria in nutrient-deprived cells [50], the rapid decrease in pH_m_ upon treatment with the decoupler CCCP inducing damage to the oxidative phosphorylation process [37], or the detection of pH_m_ bursts correlated with spontaneous decreases in mitochondrial membrane potential [48]. In our results, we focused not on the time evolution of the mitochondrial pH but on the fact that pH_m_ differences could be found between different metabophenotypes. In any case, our QD-based nanosensors fulfilled the ideal requirements for an intramitochondrial pH sensor highlighted by Santo-Domingo and Demareux: Specific targeting to the mitochondria, specific and reversible pH response, low toxicity, a wide dynamic range in the 7.6 to 8.0 pH region, and potential availability of spectral variants [48]. All these features were fulfilled by our nanosensors, including a dynamic response range between pH 6.5 and 10.0. Moreover, although we have employed herein only QDs with a single size/color, spectral variants may be easily prepared by using nanoparticles with different dimensions, hence achieving spectral variants.

The QD-SS20-MPA nanosensors were able to detect with statistical significance and reproducibility differences of pH_m_ between 0.5–0.6 units (Figure 4 and Figure 5). The highly glycolytic cell line MCF7 displayed a lower pH_m_ of 8.7, compared to the 9.2–9.3 average for the other two cell lines MDA-MD-231 and MDA-MD-468. These values were the largest levels found for intact cells. Alkaline mitochondrial matrixes, with pH_m_ values above 8.5, were reported for HeLa, NIH/3T3, MCF-7, and HBE cell lines upon treatment with H_2_O_2_, with the tumoral cells showing substantially larger pH_m_ values than that of normal cells [36]. Hence, we cannot rule out that pH_m_ values may be overestimated due to the presence of nanosensors causing enhanced ROS production. In any case, the significant pH_m_ differences found between MCF-7 and the triple-negative breast cancer cell lines evidence underlying different metabolic features. The results obtained using the pH nanosensors came to strikingly support the metabophenotypes found for the experiments performed with the metabolic inhibitors. Cancer cells displaying a high glycolytic metabolism usually show a moderately active mitochondrial oxidative metabolism. In line, a low rate/activity of mitochondrial respiration causes less proton pumping from the matrix to the intermembrane space; therefore, protons accumulate, leading to higher concentrations in the matrix than when respiration is more active. Accordingly, the higher the glycolytic dependence is, the lower the mitochondrial pH would be [48]. These preliminary studies of the metabolic behavior of the three breast cancer cell lines clearly support this hypothesis. Our current investigations will continue to establish a complete metabolic profile of the MCF7 cell line, showing glycolytic metabolism, and the MDA-MB-231 and MDA-MB-468 lines exhibiting mitochondrial oxidative phosphorylation-dependent metabolism.

## 4. Materials and Methods

### 4.1. Synthesis of SS Peptide-MPA-Capped CdSe/ZnS Nanoparticles

The synthesis of the SS peptide-MPA-capped CdSe/ZnS nanoparticles designed as intramitochondrial pH nanosensors started from commercial QDs (Mesolight, China), which were covered with a layer of hexadecylamino ligand on the surface and suspended in toluene. The first step consisted of evaporating the toluene out of a sample of 200 µL of the QD suspension by heating to 85 °C for 5 min. Next, 1.65 × 10^−3^ moles of the corresponding SS peptide were weighed and dissolved in 10 mL of MilliQ water. The 3 different SS peptides were synthesized as described in the SM (Table S1 and Schemes S1–S4). The peptide solution was added to the QDs and stirred vigorously. The mixture was sonicated for 2 h under continuous manual agitation. Then, 400 µL of MPA was added to the mixture and sonicated for 60 min. The mixture was left to react overnight. The solution was divided into 12 aliquots and centrifuged at 13,000 rpm for 10 min. After the centrifugation step, the supernatant was removed, and the pellet was resuspended in phosphate-buffered saline (PBS), pH 8. Then, an additional 100 μL of MPA was added to each aliquot and sonicated for 30 min. Finally, QD-SS-MPA nanoparticles were separated from the aqueous solution by precipitation using acetone and centrifugation, followed by the resuspension of the nanosensors in PBS, pH 8.

### 4.2. Mitochondrial Localization and FLIM Imaging of QD-SS-MPA pH Nanosensors

For the incorporation of the intramitochondrial nanosensors into live cells and the comparison of different metabolic behaviors, the following cell lines were employed: 143B (American Type Culture Collection, ATCC; CRL-8303), MCF7 (ATCC; HTB-22), MDA-MB-231 (ATCC; CRM-HTB-26), MDA-MB-468 (Leibniz-Institut DMSZ Deutsche Sammlung von Mikroorganismoen und Zellkulturen GmbH, DMSZ), and ZR751 (DMSZ).

The cells were subcultured in 6-well plates and seeded in 25 mm diameter sterilized round microscopy slides. The cells were incubated at 37 °C in 5% CO_2_ for 48 h. Prior to adding the pH nanosensors to the culture media, the QD-SS-MPA nanoparticles were sonicated for 30 min. Then, 3 µL of the stock was added to 3 mL of culture medium per well. Next, the cell plates were incubated at 37 °C for 2 h. During this time, the nanosensor penetrated into the cells and mitochondria. After incubation, the culture medium was removed, and the cells were washed twice with PBS at pH 8, taking special care to avoid cell detachment from the microscopy slides. Then, 2 mL of fresh culture medium was added to each well, with 16 µL of a 0.5 mM stock solution of the commercial mitochondria staining dye MitoTracker Deep Red (MT). Finally, the culture medium was again removed, the slides were washed twice with PBS, and fresh culture medium was placed to adequately maintain the cells at 37 °C until the start of the FLIM measurements. For FLIM analysis and pH quantifications, the slides were placed under the microscope objective with 1 mL of PBS at pH 8.

Colocalisation of the designed QD nanosensors and mitochondria and the corresponding pH studies using dual-color FLIM were performed on a MicroTime 200 FLIM system (PicoQuant). Two pulsed diode lasers, at 470 and 635 nm, were used as excitation sources and operated at a repetition rate of 10 MHz under a PIE regime. In this configuration, the 470 nm laser was used to directly excite the QD nanosensors, whereas the 635 nm laser caused direct excitation of the mitochondria-staining fluorophore MT. The collected PL emission was separated into 2 detection channels for the PL emission of the QD nanosensor (using a 520/35 bandpass filter) and the MT dye (using a 685/70 bandpass filter).

The images obtained by FLIM-PIE were analyzed using the software SyphoTime 32 (PicoQuant). The FLIM images were reconstructed by classifying all the photons corresponding to a single pixel in a temporal histogram by the time-tagged time-resolved (TTTR) methodology. Time tagging of the detected photons was performed in TimeHarp 200 modules (PicoQuant), with a time resolution of 29 ps per channel. The individual decays of each pixel were fitted to a biexponential function, with a first decay time (τ1) accounting for the autofluorescence contribution of the cell and second decay time (τ2) for the QD emission. For the measurement of pH_m_, only the pixels showing QD emission colocalized with MT fluorescence were selected. Then, the τ2 values in those pixels were transformed into a pH image using the corresponding equation from the calibration. Further details on the instrumentation and methods of analysis can be found in the SM.

## 5. Conclusions

A better understanding of tumoral metabolism may pave the way for improved classification of tumoral variants, not only based on immuno-assay and gene expression but also focusing on the biochemical pathways and dynamic homeostasis that result in high proliferation and resistance to natural defense against altered tissues. Hence, FLIM nanosensing can provide alternative tools to follow these processes at the cellular level, helping in the development of new treatments and monitoring altered metabolic states at an early stage. Our mitochondrial pH nanosensors resulted in high-throughput potential, showing high sensitivity and reproducibility to measure narrow differences of pH_m_, which interestingly support the metabolic differences found in the breast cancer cell model proposed for this work. Hence, the intramitochondrial pH could serve as a potential biomarker for detecting different metabophenotypes in breast cancer cell lines.

## Supplementary Materials

A PDF file containing the detailed methods; experimental details on the synthesis of the SS peptides; additional Figures S1–S4 showing the performance of the QD-SS31-MPA and QD-SS20-MPA nanosensors and cytotoxicity experiments; and experimental details of the viability experiments upon treatment with BMK120 and phenformin. Supplementary materials can be found at https://www.mdpi.com/1422-0067/21/10/3731/s1.

## Abbreviations

FLIM	Fluorescence lifetime imaging microscopy
QD	Quantum Dots
SS	Szeto-Schiller peptide
MPA	Mercaptopropionic acid
PL	Photoluminescence
pH_m_	Intramitochondrial pH
MT	MitoTracker Deep Red
PIE	Pulsed interleaved excitation
