# Supplementary material for: Mitochondrial pH Nanosensors for Metabolic Profiling of Breast Cancer Cell Lines
Source: Int J Mol Sci. 2020 May 25;21(10):3731. doi: 10.3390/ijms21103731 (PMC7279253; doi:10.3390/ijms21103731)
Supplement: Supplementary file 1 [file ijms-21-03731-s001.pdf]

SUPPLEMENTARY MATERIALS

Mitochondrial pH nanosensors for metabolic profiling of breast cancer cell lines

Consuelo Ripoll,[1] Mar Roldan,[2] Rafael Contreras-Montoya,[3] Juan J. Diaz-Mochon,[4] Miguel Martin,[2,5] Maria J. Ruedas-Rama,[1] Angel Orte[1,*]

[1] Departamento de Fisicoquimica. Facultad de Farmacia. Unidad de Excelencia en Quimica Aplicada a Biomedicina y Medioambiente (UEQ), Universidad de Granada, Campus Cartuja, 18071, Granada, Spain.

[2] GENYO, Pfizer-Universidad de Granada-Junta de Andalucia Centre for Genomics and Oncological Research. Avda Ilustracion 114, PTS, 18016, Granada, Spain.

[3] Departamento de Quimica Organica. Facultad de Ciencias. Unidad de Excelencia en Quimica Aplicada a Biomedicina y Medioambiente (UEQ), Universidad de Granada, Campus Fuentenueva, 18071, Granada, Spain.

[4] Departamento de Quimica Farmaceutica y Organica. Facultad de Farmacia. Unidad de Excelencia en Quimica Aplicada a Biomedicina y Medioambiente (UEQ), Universidad de Granada, Campus Cartuja, 18071, Granada, Spain.

[5] Departamento de Bioquimica y Biologia Celular I. Facultad de Ciencias. Universidad de Granada, Campus Fuentenueva, 18071, Granada, Spain.

TABLE OF CONTENTS

Supplementary experimental section **2**
Instrumentation *2*
Analysis of the FLIM images *2*

Synthesis of Szeto-Schiller peptides **3**
Table S1. Reagents used in each stage of the solid-phase synthesis of the SS peptides. *4*
Scheme S1. Synthesis of the Rink polystyrene resin. *5*
Scheme S2. Synthesis of the SS20-mercapto peptide: $HS\text{-}(CH_2)_{10}\text{-}CONH\text{-}Phe\text{-}Arg\text{-}Phe\text{-}Lys\text{-}CONH_2$. *5*
Scheme S3. Synthesis of the SS02-mercapto peptide: $HS\text{-}(CH_2)_{10}\text{-}CONH\text{-}Dmt\text{-}Arg\text{-}Phe\text{-}Lys\text{-}CONH_2$. *6*
Scheme S4. Synthesis of the SS31-mercapto peptide: $HS\text{-}(CH_2)_{10}\text{-}CONH\text{-}Arg\text{-}Dmt\text{-}Lys\text{-}Phe\text{-}CONH_2$. *7*

Cellular localization of the QD-SS31-MPA nanosensor **8**
Figure S1. *Representative dual-color images of the QD-SS31-MPA (green channel) and the MT staining dye (red channel) in 143B cells.* *8*

Supplementary figures of the performance of the QD-SS20-MPA nanosensor **9**
Figure S2. *Additional representative colocalization images of the QD-SS20-MPA and the MT staining dye in different cell lines.* *9*
Figure S3*. Additional representative pH images, obtained from the PL lifetime, τ, of QD-SS20-MPA nanosensors incorporated into the mitochondria of MCF7 (**a**), MDA-MB-231 (**b**), and MDA-MB-468 (**c**) breast cancer cell lines.* *10*

Cytotoxicity of the nanosensors **11**
Figure S4. *Cell viability and cytotoxicity of QD-SS20-MPA nanosensors.* *11*

Effect of phenformin and BMK120 on cellular metabolism **12**

Supplementary references **12**

SUPPLEMENTARY EXPERIMENTAL SECTION

Instrumentation

Steady-state photoluminescence (PL) spectra and PL decay traces to check the pH response of the quantum dot-Szeto Schiller (SS) peptide-mercaptopropionic acid (QD-SS-MPA) nanosensors were collected on an FP-8300 spectrofluorometer (Jasco) and a FluoTime200 time-resolved spectrofluorometer (PicoQuant), respectively. Dynamic light scattering (DLS) was performed on a Zetasizer μV (Malvern). Transmission electron microscopy (TEM) images were collected using a LIBRA 120 PLUS (Carl Zeiss SMT) at the Centro de Instrumentacion Cientifica-Universidad de Granada (CIC-UGR).

Cell viability experiments were performed using the fluorogenic CellTiter Blue test by measuring the fluorescence in a Glo-Max®-Multi+ Detection System (Promega).

Colocalization of the designed QD nanosensor and mitochondrial organelles and the corresponding pH studies using dual-color fluorescence lifetime imaging microcopy (FLIM) were performed on a MicroTime 200 FLIM system (PicoQuant). Two pulsed diode lasers, at 470 and 635 nm, were used as excitation sources, operated at a repetition rate of 10 MHz, with the 635-nm laser pulsed being delayed by 56 ns to reach the pulsed interleaved excitation (PIE) regime. The laser power at the microscope entrance was between 0.2 and 4.4 μW for both lasers. In this configuration, the 470-nm laser was used to directly excite the QD nanosensors, whereas the 635-nm laser caused direct excitation of the mitochondria-staining fluorophore MitoTracker Deep Red (MT). The PIE excitation scheme allows us to define photon detection time windows to reduce artifacts due to cross-talk between channels [1]. The excitation laser beams passed through an achromatic quarter-wave plate (AQWP05M-600, Thorlabs) set at 45º from the polarization plane of the laser and were directed by a specific dual-band dichroic mirror (AHF/Chroma), prepared for 470/635 dual excitation, into the specimen through the apochromatic oil immersion objective (100 ×, 1.4NA). The collected PL emission was focused into a 75-μm pinhole after passing through an LP500 longpass filter (AHF/Chroma). Finally, the emitted light was separated into two SPAD detection channels using a 600DCXR (AHF/Chroma) dichroic mirror. The first channel was dedicated to the tested QD nanosensor using a 520/35 bandpass filter (AHF/Chroma), whereas the second channel was solely dedicated to the red fluorescence from the MT using a 685/70 bandpass filter (AHF/Chroma). Time tagging of the detected photons was performed in TimeHarp 200 modules (PicoQuant), with a time resolution of 29 ps per channel. Images were collected with a 512 × 512 pixel resolution and a collection time of 0.60 ms per pixel.

Analysis of the FLIM images

The images obtained by FLIM-PIE were analyzed using the software SyphoTime 32 (PicoQuant). FLIM images were reconstructed by classifying all the photons corresponding to a single pixel in a temporal histogram by the time-tagged time-resolved (TTTR) methodology. The regions of interest were selected (the pixels that contained the QD emissions and that had at least 200 photons per pixel); in this way, the global PL decay of these regions was obtained. The decay was adjusted with a biexponential decay function and was subjected to the iterative reconvolution method based on the maximum likelihood estimator (MLE), which is a more reliable method for decays with low counting rates. The instrument response function (IRF) for the iterative reconvolution analysis was obtained by reconstruction from an experimental PL decay of an image with a large number of photon counts. To obtain the FLIM image, a spatial binning of 5 × 5 pixels and a temporal binning of 4 time channels (for a final temporal resolution of 116 ps/channel) were applied to obtain higher counts in each pixel. The individual decays of each pixel were fitted to the same multiexponential function, leaving the first decay time ($\tau 1$) as a fixed parameter with a value of 1.5 ns, which accounts for the auto-fluorescence contribution of the cell, and the second decay time ($\tau 2$) and the pre-exponential factors as adjustable parameters. Finally, the image containing the value of the adjustable lifetime ($\tau 2$) in each pixel was transformed into a pH image using the corresponding equation from the calibration described in the main text. The images shown in Figures 3 and S3 as well as the corresponding pH distributions account for only those pixels that are colocalized with MT fluorescence in the red channel, surpassing a specific intensity threshold value. The selection of pixels of interest and the reconstruction of $\tau 2$ and pH distributions have been implemented in home-coded scripts in MathCad 15.0 (PMC).

SYNTHESIS OF SZETO-SCHILLER PEPTIDES

The synthesis of the SS peptides was performed within the context of the collaboration between the company Nanogetic S.L. (Granada, Spain) and our research group. The company provided the final materials after the synthesis reactions were optimized. Since this optimization is part of the research project, it is described in these Supplementary Materials.

Coupling reactions were carried out in the solid phase using 10 mL of solvent per gram of resin. To test the coupling reactions, a qualitative ninhydrin test was carried out after each reaction, as described in the literature [2].

The solid-phase synthesis of the peptides was carried out using the reagents listed in Table S1 and a series of common protocols described below. The first step was obtaining the modified Rink polystyrene resin (Scheme S1). Then, serial addition of the corresponding reagents resulted in the final synthesis of the SS peptides. Three different SS peptides were prepared, containing an 11-carbon aliphatic chain finishing on a thiol group at their N-terminal end. Conjugation with the QD surface thus occurred via formation of self-assembly monolayers between thiol groups and metallic surfaces.

Standard solid-phase peptide synthesis (SPPS) protocols were used, briefly:

Method A – Solid-phase peptide coupling using carboxylic acids and free primary amines: The resin was previously swollen in dimethylformamide (DMF). Then, carboxylic acid (3.5 equiv.) and hydroxybenzotriazole (HOBt) (5 equiv.) were dissolved in DMF. Diisopropylcarbodiimide (DIC) (5 equiv.) was added, and the mixture was stirred for 15 min before addition to the resin. The mixture was agitated for 2 h at 60 °C to favor amide formation. The resin was then washed with DMF (×5), dichloromethane (DCM) (×5) and diethyl ether (×2).

Method B – Capping, free amine acetylation: The resin was suspended in DMF. Then, pyridine (2 equiv.) and acetic anhydride (2 equiv.) were added to the suspension. The mixture was agitated for 15 min. The resin was washed as in Method A.

Method C – *N*-terminal Fmoc removal: Fmoc removal was performed using 20% piperidine in DMF with two sequential 20-min treatments. The resin was then filtered and washed with DMF (×5), DCM (×5) and diethyl ether (×2).

Method D – cleavage of peptides from resin and acid-labile protecting group removal: Dry resin was suspended in 20 mL per gram of TFA/H_2O/EDT/TIS (93.5/2.5/2.5/1.5) resin and agitated for 2 h. The TFA solution was collected, reduced by vacuum and then the remaining TFA solution was added drop wise to cold (4 °C) diethyl ether in a centrifuge tube. The resulting precipitate was finally collected by centrifugation and washed with diethyl ether (×3), giving rise to white powders.

The synthesis reactions for the Rink polystyrene resin and the mercapto-modified SS20, SS02 and SS31 peptides, which incorporate the established common procedures described above, are depicted in Schemes S1, S2, S3, and S4, respectively.

Table S1. Reagents used in each stage of the solid-phase synthesis of the SS peptides.

Reagent code	Carboxylic acid as reagent	Amino acid coupled
**I**	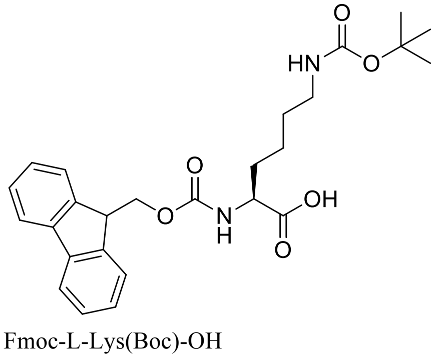 Fmoc-L-Lys(Boc)-OH	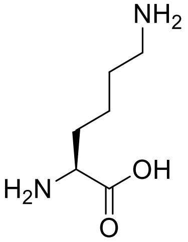 L-Lysine
**II**	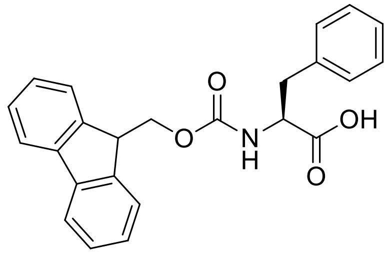 Fmoc-L-Phe-OH	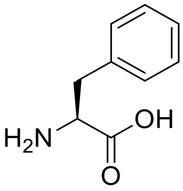 L-Phenylalanine
**III**	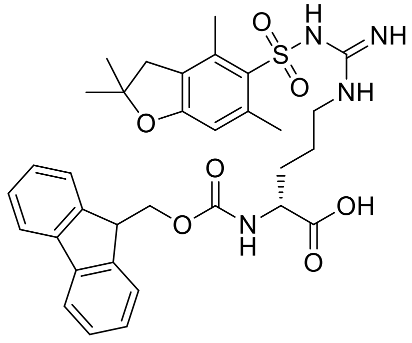 Fmoc-D-Arg(Pbf)-OH	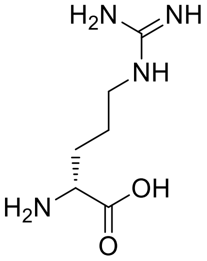 D-Arginine
**IV**	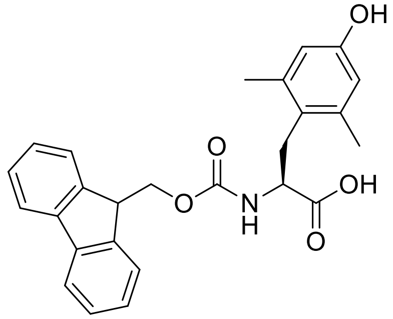 Fmoc-L-Dmt-OH	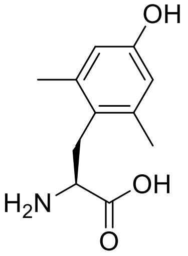 L-Dimethyltyrosine
**V**	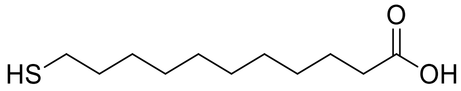 Mercaptoundecanoic acid	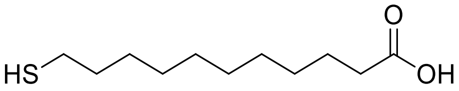 Mercaptoundecanoic acid

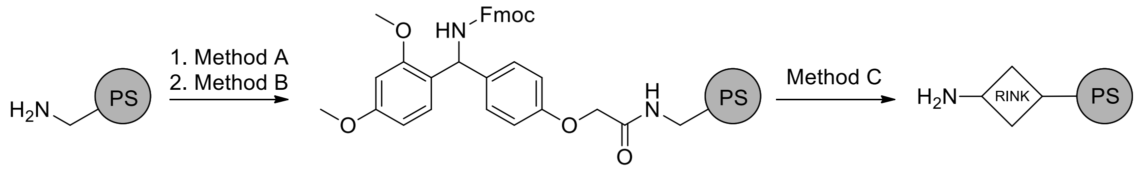

Scheme S1. Synthesis of the Rink polystyrene resin.

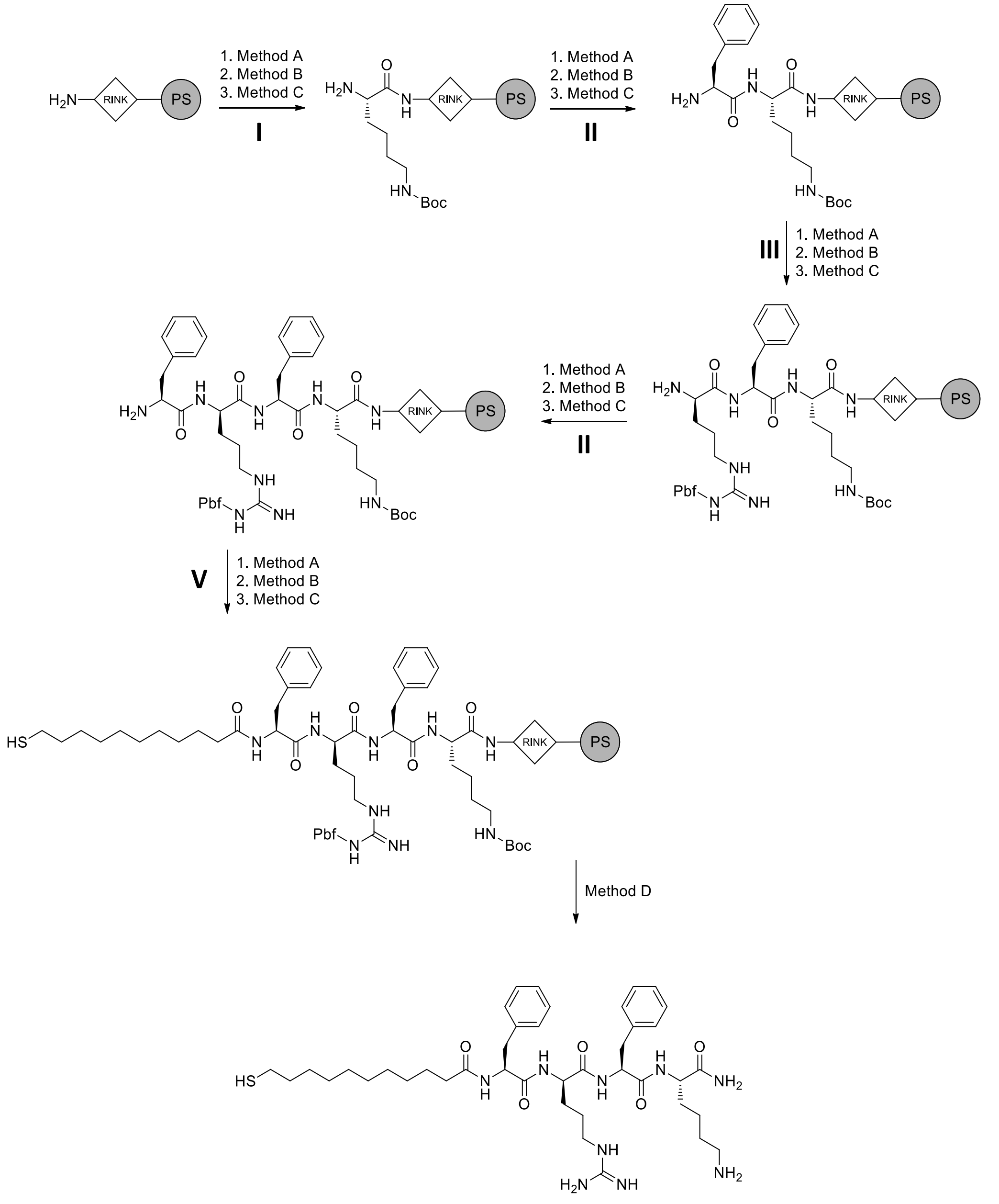

Scheme S2. Synthesis of the SS20-mercapto peptide: HS-$(CH_2)_{10}$-CONH-Phe-Arg-Phe-Lys-$CONH_2$.

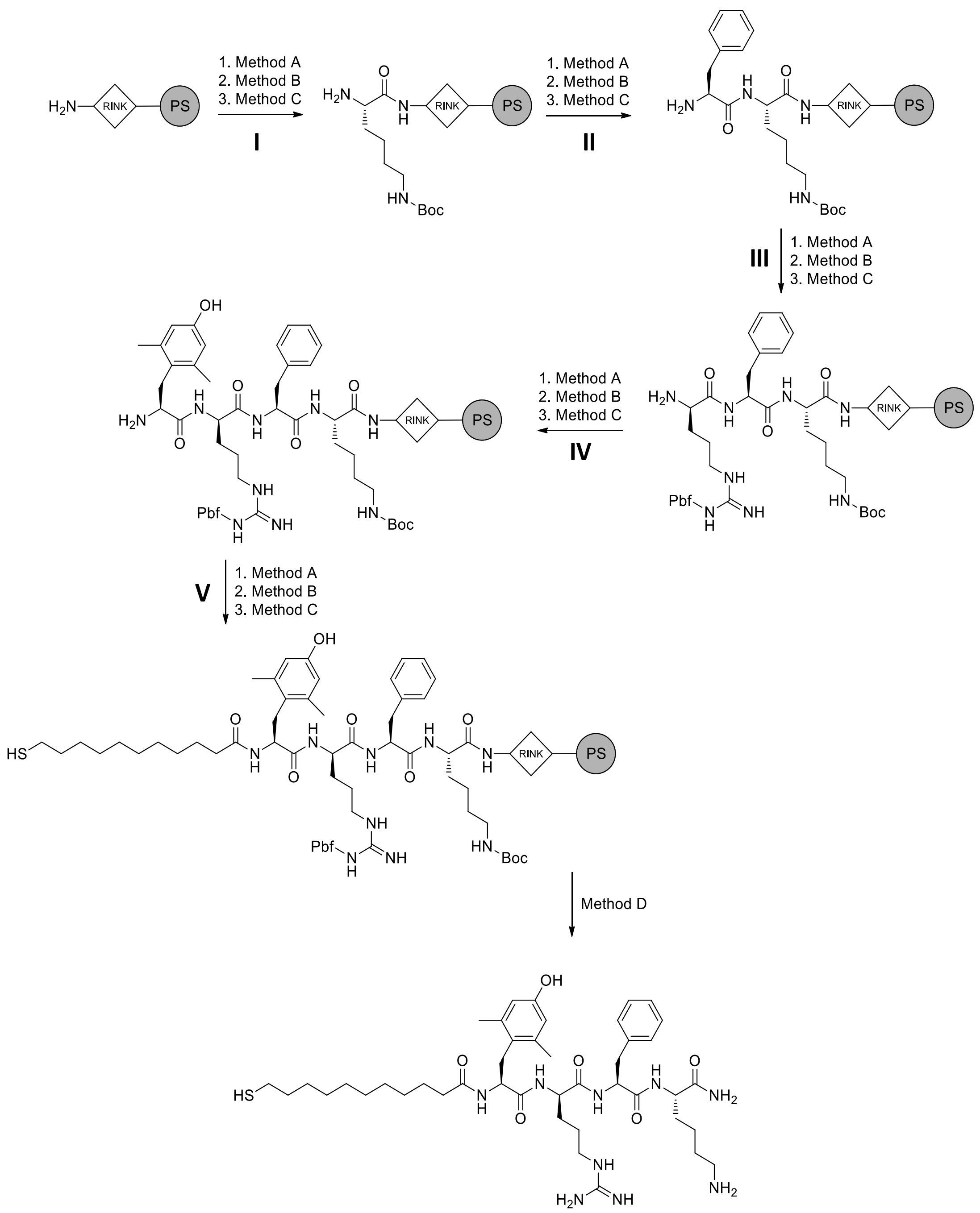

Scheme S3. Synthesis of the SS02-mercapto peptide: HS-$(CH_2)_{10}$-CONH-Dmt-Arg-Phe-Lys-$CONH_2$.

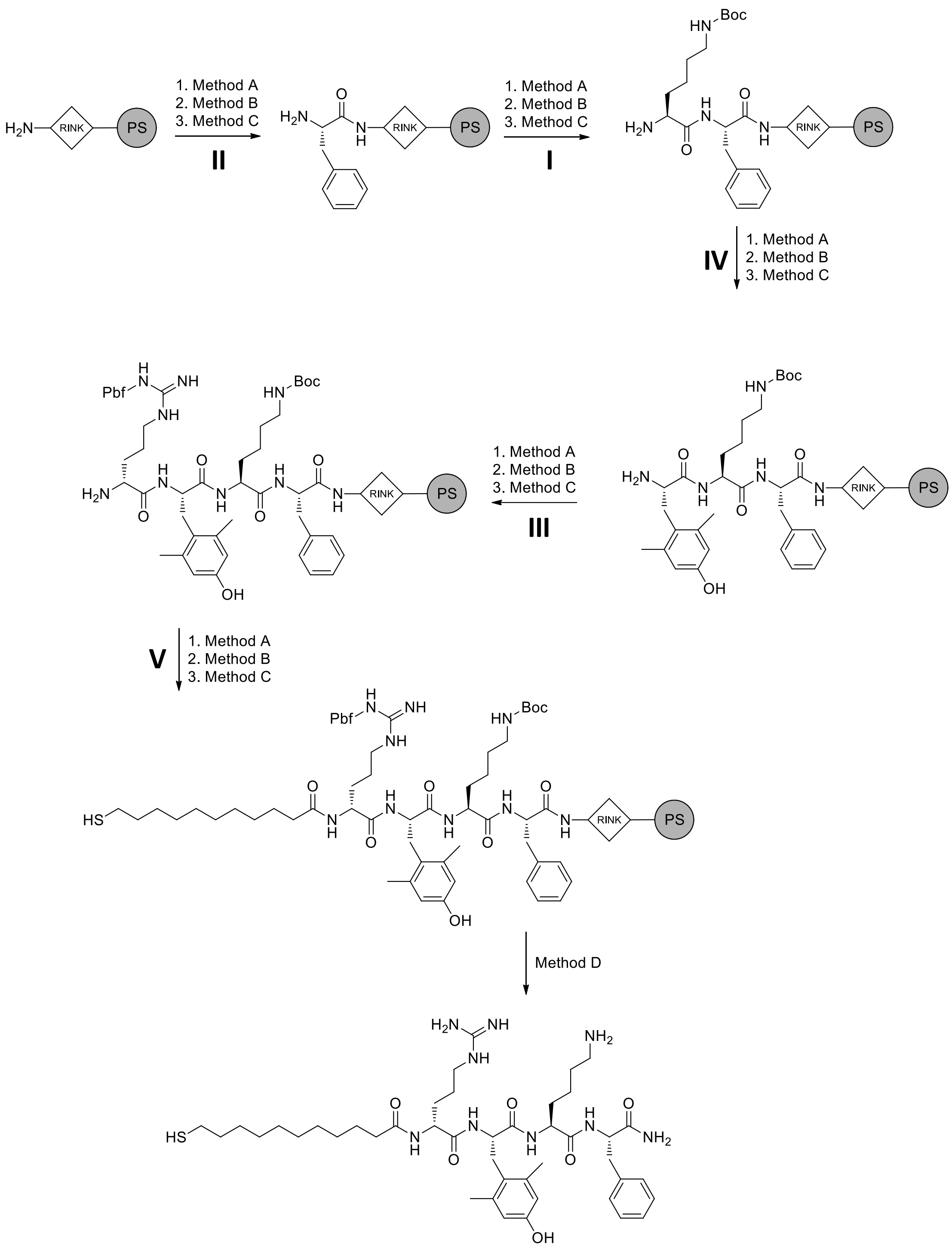

Scheme S4. Synthesis of the SS31-mercapto peptide: HS-$(CH_2)_{10}$-CONH-Arg-Dmt-Lys-Phe-$CONH_2$.

CELLULAR LOCALIZATION OF THE QD-SS31-MPA NANOSENSOR

QD-SS31-MPA nanoparticles were prepared as detailed in the experimental section of the main text. The experiments to study the mitochondrial intake of these particles were also performed as described. Figure S1 shows that, in contrast to what was expected, the QD-SS31-MPA nanosensor did not colocalize with the mitochondria. Therefore, we discarded this construct in subsequent studies.

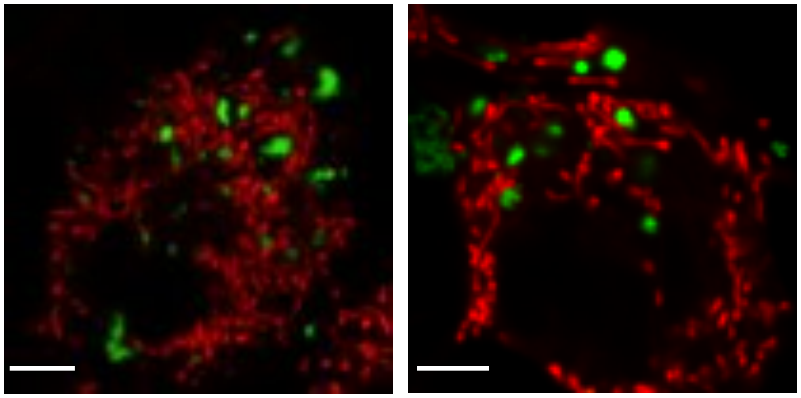

Figure S1. Representative dual-color images of the QD-SS31-MPA (green channel) and the MT staining dye (red channel) in 143B cells.

Scale bars represent 10 μm.

SUPPLEMENTARY FIGURES OF THE PERFORMANCE OF THE QD-SS20-MPA NANOSENSOR

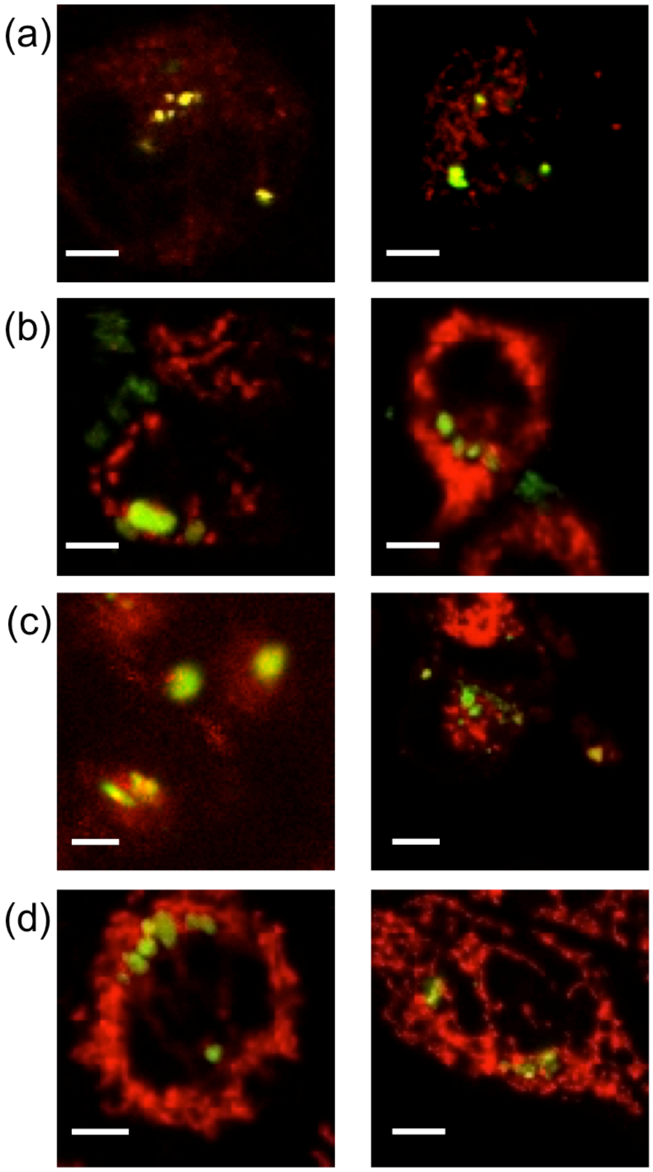

Figure S2. Additional representative colocalization images of the QD-SS20-MPA and the MT staining dye in different cell lines.

The figure shows representative colocalization images of the QD-SS20-MPA (green channel) and the MT staining dye (red channel) in 143B osteoblasts (**a**), and MCF7 (**b**), MDA-MB-231 (**c**), and MDA-MB-468 (**d**) breast cancer cell lines. The images were selected from a set of fifteen different images for each cell line. Scale bars represent 10 μm.

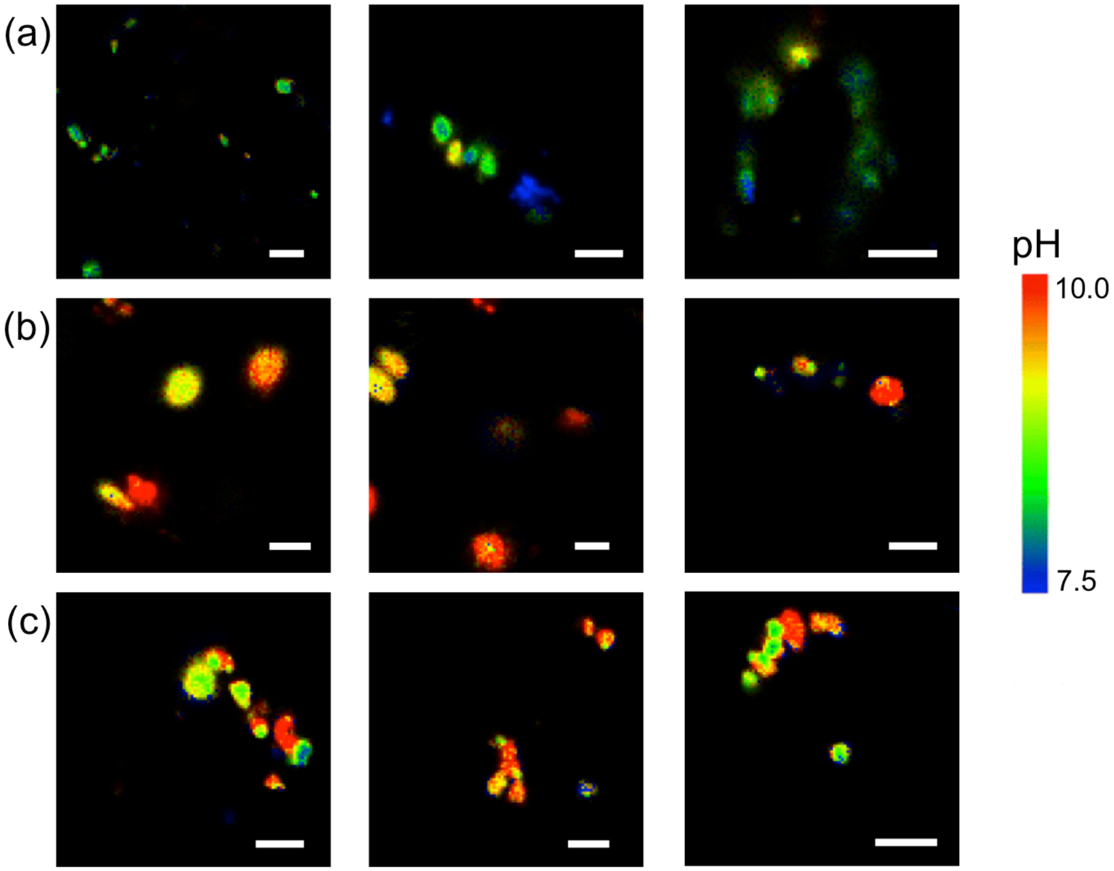

Figure S3. Additional representative pH images, obtained from the PL lifetime, τ, of QD-SS20-MPA nanosensors incorporated into the mitochondria of MCF7 (**a**), MDA-MB-231 (**b**), and MDA-MB-468 (**c**) breast cancer cell lines.

Scale bars represent 10 μm

CYTOTOXICITY OF THE NANOSENSORS

The QDs used in this work consist of a core of CdS with an external layer of ZnS. Therefore, as an important control, it was essential to rule out that QDs would affect cell viability. Some studies published several years ago found that QDs were toxic in biological samples [3-5]. Because of these studies, we tested the cytotoxicity of the QDs in two of the breast cancer cell lines used in this work (MCF7 and MDA-MB-468).

The cells were incubated for two hours with three different amounts of QD (1 µL, 2 µL, and 4 µL per mL of culture medium), and cell viability was measured using CellTiter Blue. This test is based on the ability of living cells to convert one nonfluorescent molecule (resazurin) into another fluorescent molecule (resorufin). To carry out these experiments, the cell lines were seeded in 96-well plates, and the QD-SS-MPA nanosensors were added. Then, the plates were incubated at 37 °C for 2 h, 6 h and 24 h. After incubation, 20 µL of CellTiter Blue reagent was added per 100 µL of culture medium to each well, and then the wells were incubated for 20 min at 37 °C. Finally, the fluorescence was measured in a GloMax®-Multi+ Detection System (Promega).

Starting from the amount of QDs used in the main experiments for mitochondrial colocalization and intramitochondrial pH measurements, we also doubled and quadrupled this dose of QDs to test higher concentrations of QD delivery. Likewise, the incubation time of QD in cells for the experiments described in the main text was 2 h, but we also performed additional viability tests with longer incubation times of 6 h and 24 h. For these tests, 1 µL of the stock QD solution per mL of culture medium was used. As seen in Figure S4, cell viability was not significantly affected in any of the treatments tested, including the treatments with high concentrations of QDs (panel **a**) and those with long incubation times (panel **b**). Therefore, these QDs can be considered suitable for use with biological samples, at least under the conditions employed in our experiments.

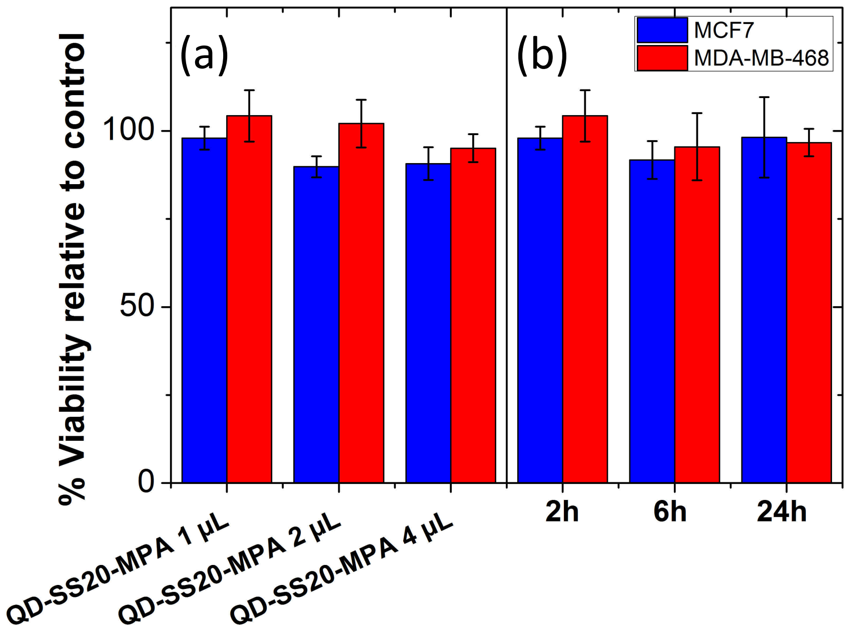

Figure S4. Cell viability and cytotoxicity of QD-SS20-MPA nanosensors.

The figure shows the results of experiments testing the viability of MCF7 and MDA-MB-468 cells in the presence of QD-SS20-MPA at three different doses and incubated for 2 h (**a**), or after three different incubation times at a dose of 1 µL/mL of culture medium (**b**).

EFFECT OF PHENFORMIN AND BMK120 ON CELLULAR METABOLISM

Additional experiments were designed to test the impact on cell viability of inhibitors for selected metabolic targets. The aim was to test whether the breast cancer subtypes represented by the different cell lines display a differential or similar response to key metabolic targets. Two experiments were performed to address possible differences concerning major metabolic features, between the breast cancer cell lines representative of the clinical subtypes. In line, experiments using metabolic inhibitors were performed, aiming to answer whether the overall metabolism of these cells mainly relies on a high glycolytic dependence. We focused on the dependence of the glycolysis metabolism, and performed cell viability experiments. Cell viability experiments were carried out with cells cultured in black 96-well plates, using a high-throughput fluorescence assay. The CellTiter Blue cell viability assay (Promega) was chosen to test the impact of the selected drugs on cell viability. CellTiter Blue is an assay based on the ability of living cells to convert resazurin (a redox dye) into resorufin (a fluorescent product). When cells die, they lose the capability to carry out this reaction. Thus, cell viability can be quantified by fluorescence in real time for living cells.

Cells were seeded in the wells with the specified culture medium. The MCF7 cells were seeded at a density of 1×10^4, and the MDA-MB-231 and MDA-MB-468 cells were seeded at a density of 8×10^3 in 100 µL of culture medium. After plating, the inhibitors or drugs were added, and the plate was maintained for 96 h in an incubator at 37 °C. After 96 h of treatment, 20 µL of CellTiter Blue reagent was added per 100 µL of culture medium to each well. Then, the cells were incubated for 20 min at 37 °C, and the fluorescence was measured in a GloMax®-Multi+ Detection System (Promega). The average of all replicates was calculated, and then the value of the blank was subtracted from the average. The data were expressed as percentages relative to control samples, and the control values were assigned as 100%.

The first treatment was the use of phenformin, a biguanide commonly used as therapy for type 2 diabetes but recently described as a potential anticancer molecule due to its ability to inhibit the mitochondrial complex I [6]. This compound inhibits the reactions of substrate oxidation in mitochondrial respiratory complex I, therefore drastically reducing the conversion of NADH to NAD^+ and affecting the cellular redox balance and, in particular, the availability of NAD^+ to maintain the rate of glycolytic activity and the synthesis of aspartate. Cells were seeded with or without the addition of 50 µM phenphormin. After 96 h, the cell viability was measured to test the effect and response towards phenphormin. The MCF7 cell line was sensitive, displaying a decrease in cell viability to 30-40%. In contrast, the MDA-MB-231 and MDA-MB-468 cells lines were considered resistant, as both cell lines maintained almost 100% viability. Therefore, the breast cancer cell line MCF7 showed high dependence on NAD^+ availability. These results can be seen in Figure 5b in the main text.

We next tested the response to a direct enzymatic deregulation of the glycolytic pathway. For this purpose, the inhibition of the Akt pathway was chosen to induce a decrease in glycolytic flux. Akt is a serine/threonine kinase. The inhibition of Akt leads to a direct deregulation of some of the enzymes of the glycolytic pathway, blocking one of the main glycolytic activation pathways. Thus, we performed experiments to test the effect of the Akt inhibitor BKM120. Cells were seeded with or without the addition of 200 µM BKM120, and the cell viability was measured after 96 h at 37 °C [7]. The results indicated that there were two different metabophenotypes for the glycolytic dependency of the breast cancer cell subtypes. The data showed a different metabophenotype for MCF7, which relied on an active glycolytic flux and was sensitive to BKM120-induced Akt inhibition, displaying 45% cell viability. On the other hand, the MDA-MB-231 and MDA-MB-468 cell lines presented a resistant metabophenotype, displaying values of cell viability above 70-75%. These results can be seen in Figure 5c in the main text.

Interestingly, the results clearly displayed two distinct glycolytic phenotypes, so-called metabophenotypes (Figure 5). MCF7 cells displayed a stronger dependence on glycolytic pathways, since both, NAD^+ imbalance due to phenformin inhibition on respiration complex I (Figure 5b in the main text) and Akt inhibition due to BKM120 treatments (Figure 5c in the main text) provoked a significant loss of cell viability. In contrast, MDA-MD-231 and MDA-MB-468 resulted almost resistant to drug treatments, thus suggesting a low glycolytic dependence.

SUPPLEMENTARY REFERENCES

1. Müller, B. K.; Zaychikov, E.; Bräuchle, C.; Lamb, D. C., Pulsed Interleaved Excitation. *Biophys. J.* **2005,** 89, (5), 3508-3522.
2. Kaiser, E.; Colescott, R. L.; Bossinger, C. D.; Cook, P. I., Color test for detection of free terminal amino groups in the solid-phase synthesis of peptides. *Anal. Biochem.* **1970,** 34, (2), 595-598.
3. Bradburne, C. E.; Delehanty, J. B.; Boeneman Gemmill, K.; Mei, B. C.; Mattoussi, H.; Susumu, K.; Blanco-Canosa, J. B.; Dawson, P. E.; Medintz, I. L., Cytotoxicity of Quantum Dots Used for In Vitro Cellular Labeling: Role of QD Surface Ligand, Delivery Modality, Cell Type, and Direct Comparison to Organic Fluorophores. *Bioconjugate Chem.* **2013,** 24, (9), 1570-1583.
4. Winnik, F. M.; Maysinger, D., Quantum Dot Cytotoxicity and Ways To Reduce It. *Acc. Chem. Res.* **2013,** 46, (3), 672-680.
5. Chen, N.; He, Y.; Su, Y.; Li, X.; Huang, Q.; Wang, H.; Zhang, X.; Tai, R.; Fan, C., The cytotoxicity of cadmium-based quantum dots. *Biomaterials* **2012,** 33, (5), 1238-1244.
6. Weinberg, S. E.; Chandel, N. S., Targeting mitochondria metabolism for cancer therapy. *Nature Chem. Biol.* **2014,** 11, 9.
7. Jiang, Z.; Huang, J.; Xie, C.; Li, X.; Liu, L.; He, J.; Pan, H.; Huang, L.; Fan, X.; Yao, X.; Xie, Y.; Li, N.; Liu, L.; He, J.; Leung, E. L., Combined use of PI3K and MEK inhibitors synergistically inhibits lung cancer with EGFR and KRAS mutations. *Oncol. Rep.* **2016,** 36, (1), 365-375.
